# Understanding the Global Problem of Drug Addiction is a Challenge for IDARS Scientists

**DOI:** 10.2174/157015911795017245

**Published:** 2011-03

**Authors:** S.F Ali, E.S Onaivi, P.R Dodd, J.L Cadet, S Schenk, M.J Kuhar, G.F Koob

**Affiliations:** 1 Neurochemistry Laboratory, NCTR/FDA, Jefferson, AR, USA; 2 William Paterson University, Wayne, NJ, USA; 3 Molecular Neurobiology Branch, National Institute on Drug Abuse, NIH, Baltimore, MD, USA; 4 University of Queensland, Brisbane, Australia; 5 Molecular Neuropsychiatry Branch, NIDA/NIH/DHHS, Baltimore, MD, USA; 6 Victoria University of Wellington, Wellington, New Zealand; 7 Yerkes National Primate Research Center of Emory University, Atlanta, GA, USA; 8 The Scripps Research Institute, La Jolla, CA, USA

**Keywords:** International Drug Abuse Research Society, IDARS, addiction, alcohol, marijuana, psychostimulants.

## Abstract

IDARS is an acronym for the International Drug Abuse Research Society. Apart from our scientific and educational purposes, we communicate information to the general and scientific community about substance abuse and addiction science and treatment potential. Members of IDARS are research scientists and clinicians from around the world, with scheduled meetings across the globe. IDARS is developing a vibrant and exciting international mechanism not only for scientific interactions in the domain of addiction between countries but also ultimately as a resource for informing public policy across nations. Nonetheless, a lot more research needs to be done to better understand the neurobiological basis of drug addiction – A challenge for IDARS scientists.

## INTRODUCTION

Drug addiction is a chronically relapsing disorder that has been characterized by the compulsive use of addictive substances despite adverse consequences to the individual and society [1]. Addiction to drugs and alcohol is increasingly becoming a worldwide trend in lifestyle that is prevalent in rich and poor countries alike. Addiction to alcohol, drugs and cigarette smoking is now regarded as a major public health problem. Other forms of addiction including computer games, gambling, sex and food also have severe consequences on the health of the individual and to society. The commonly abused drugs have profound action in the nervous system, particularly in the brain. Some of these substances such as opium, marijuana, cocaine, nicotine, caffeine, mescaline, and psilocybin are obtained from natural sources while others are synthetic or designer drugs. Furthermore some of these substances like alcohol and nicotine are legal while some others that are legally available by prescription have addictive potential in vulnerable individuals. A number of addictive substances are illegal in most countries and this fuel the illegal drug trafficking and business that are often associated with criminal activities. The initiation of the use of these substances induces euphoria, reward and a state of well-being that can lead to physical and psychological dependences. Withdrawal syndrome occurs when the individual attempts to stop the use of addictive substances and this leads to the cycle of dependency. The mechanism(s) associated with the cycle of addiction include neuronal adaptation with tolerance or sensitization involved in the action of addictive substances. A number of factors have also been associated with addiction, including the availability, cost, method of administration, environmental factors such as behaviors acceptable in a community, peer influences and genetic and epigenetic factors. Over the years a number of therapeutic approaches for drug and alcohol addiction have been utilized. However, relapse the resumption of drug taking following a period of drug abstinence, is considered the main hurdle in treating drug addiction. Unfortunately pharmacological treatment of drug and alcohol dependency has largely been disappointing and new therapeutic targets and hypotheses are needed. Drug addiction is also influenced by the interaction of genes, epigenetics and the environment. Twin studies consistently show that there is a heritable component to drug abuse and addiction [2]. Now using modern genomic techniques, we are able to examine genetic variants, or single nucleotide polymorphisms that contribute to addiction vulnerability. So a lot more research needs to be done to better understand the neurobiological basis of drug addiction and hence a continuous challenge for IDARS scientists. IDARS is therefore engaged in a vibrant and exciting international mechanism, not only for scientific interactions among scientists in the domain of addiction research between countries but also as a resource for informing public policy across nations. This is an exciting period in the study of the neurobiology of addiction where brain circuitry and molecular mechanisms are providing hope for understanding not only the vulnerability to addiction but also providing new targets for the treatment of various types of substance abuse/dependence as presented in this report.

## MARIJUANA HIGHLIGHTS

Various forms of marijuana preparations comes from the cannabis plant and is the most commonly used drug in the world, for recreation and suddenly, we are awakened to potential therapeutic applications. Therefore, these are high times for marijuana research with new findings on the biological effects of cannabinoids and as new potential applications in neurological and neuropsychiatric disorders [3]. The new advances and understanding indicate that the cellular, molecular and behavioral responses to marijuana are encoded in our genes [3]. The discovery that specific genes codes for cannabinoid receptors (CBRs) that are activated by marijuana use, and that the human body makes its own marijuana-like substances - endocannabinoids [4], that also activates CBRs have provided surprising new knowledge about cannabinoid genomic and proteomic profiles. These remarkable advances in understanding the biological actions of marijuana, cannabinoids and endocannabinoids, is unraveling the genetic basis of marijuana use and the implication in human health and disease. We know that the two well characterized cannabinoid CB1 and CB2 receptors are encoded by *CNR1* and *CNR2* genes that have been mapped to human chromosome 6 and 1 respectively. A number of variations in cannabinoid receptor genes have been associated with human disorders including drug dependency [4], osteoporosis [5], ADHD and PTSD, [6, 7], obesity [8, 9], and depression [10, 11]. Thus, because of the ubiquitous distribution and role of the endocannabinoid system in the regulation of a variety of normal human physiology, drugs that are targeted to different aspects of this system are already benefiting cancer subjects and those with AIDs and metabolic syndromes [8]. In the coming era of personalized medicine, genetic variants and haplotypes in *CNR1* and *CNR2* genes associated with obesity or addiction phenotypes may help identify specific targets in conditions of endocannabinoid dysfunction. Most strikingly, variants of *CNR* genes co-occur with other genetic variations and share biological susceptibility that underlies comorbidity in many neuropsychiatric disturbances [12]. Therefore, understanding the endocannabinoid system in the human body and brain will contribute to elucidating this natural regulatory mechanism in health and disease.

## CHRONIC ALCOHOLISM IN HUMAN SUBJECTS

Illicit drug use has well-known detrimental effects, but the legal drugs tobacco and alcohol have far greater impact on human health world-wide. By a standard measure of morbidity, dalys, tobacco is at 4.1%, alcohol 4.0% (6.6% for males, 3.1% for females [13]); all illicit drugs combined add only 0.8% to global disease burden [14]. Alcohol consumption is common: in one survey, 82% of respondents over 14 had imbibed it in the previous 12 months [15]. The social cost of alcohol use is also very high [16, 17]. Alcohol misuse leads to selective brain pathology, though subjects differ markedly [18]. Brain shrinkage, reduced white-matter volume, and dendritic pruning may be reversible with abstinence; irreversible effects are more focal: superior prefrontal cortex (sfc) is particularly vulnerable [19, 20]. Common comorbidities (cirrhosis of the liver and the Wernicke-Korsakoff syndrome), which are more prevalent with higher rates of alcohol consumption [21], lead to greater brain atrophy [18]. Neuronal death may be due to *excitotoxicity*, an imbalance between excitatory and inhibitory inputs mediated by NMDA receptors (NMDAR) and reduced GABA_A_-mediated responses [22, 23]. This *allostasis* [24, 25] maintains a proper balance between excitation and inhibition in the presence of alcohol. When alcohol is removed, as in periods of abstinence, the balance is skewed towards over-excitation. This leads to a large influx of Ca^2+^ ions into cells, affecting many signaling pathways and eventually leading to cell death. In rat and mouse models NMDAR are increased after chronic alcohol, but there are regional and species differences due to modes of administration, age, time of withdrawal, etc [26-29]. All studies show increases in at least one NMDAR subunit [30-34]. However, the area affected and the subunit(s) that change are paradigm-dependent [30, 32].

In human autopsy studies, NMDAR are increased in sfc and hippocampus [35, 36]. Alcoholics without comorbid disease shift the excitatory balance by GABA_A_ receptor subunit switching to reduce inhibitory function. GABA_A_ shifts are small in cirrhotic alcoholics, and the key change may be increased excitability *via* altered NMDAR expression. Using quantitative real-time PCR we found that alcoholics without liver cirrhosis did not differ significantly from controls in the expression of any subunit, whereas all subunits were significantly lower in cirrhotic alcoholics [37-40]. Promising NMDAR-specific tracers for PET and SPECT provide the potential to study NMDAR changes in human subjects in the future [41-43].

Chronic alcohol misuse affects the expression of many genes in the brain, leading to long-term changes in neural function. Microarray and proteomic studies have found changes in the expression of genes involved in metabolism, immune response, cell survival, cell communication, signal transduction, and energy production, DNA-binding proteins, transcription factors, repair enzymes, myelination, and cell-adhesion [44-46].

### The Genetics of Alcoholism

Alcoholism in human subjects is mediated by many societal and genetic factors. Genes may mediate etiology and pathogenesis, although this issue is still hotly debated. Different genetic markers are associated with increased risk of alcohol misuse, dependence, craving, tolerance, and withdrawal severity [47]. Risk-factor genes code for alcohol-metabolising enzymes, and also for the effectors of neurotransmission – receptors, transporters and signal-transduction components – for a variety of transmitter classes, including dopamine, serotonin, glutamate, and GABA. Some genes are associated with general aspects of addiction [48, 49].

The effects of these polymorphisms can be divided into two categories: those that pre-dispose the individual to alcohol abuse, and those that make an individual more susceptible to the toxic effects of alcohol. Polymorphisms may not only alter the product of the parent gene (e.g., by changing the amino-acid codon) but may also have pleiotropic effects, i.e., the changes in one gene may affect the expression of, or activity of the product of, another gene. An emerging microarray literature is showing that knocking out a single gene alters the expression of hundreds of transcripts, many of which have no discernible association with the knocked-out gene. Knockout mice strains that differ in alcohol responsiveness have been compared to find transcripts that show discriminant expression [50]. Affected transcripts are from genes located on a range of chromosomes – not only that bearing the knocked-out gene. It is difficult to transfer this concept to human brain but an analogue of a gene knockout relevant to human alcoholism is the *ALDH2* gene (*ALDH2*-2,2 homozygotes have no ALDH activity [51]). More generally, allelic variants of alcoholism-associated genes are very likely to moderate the expression of a range of genes. There is a multiplier effect for expression whereby proteins usually show larger effect sizes than mRNA transcripts [52]: proteins are more readily linked to functional differences than transcripts.

### Dopamine and Alcohol

Dopamine (DA) is a monoamine transmitter that mediates motivation, attention, short-term memory and reinforcement. Many dopaminergic neurones originate in the ventral tegmental area (VTA) and project to the cerebral cortex, nucleus accumbens (NAc) and amygdala [53]. The mesolimbic system of the brain has been a focus of addiction research because it involves the so-called pleasure-centers such as the NAc. Dopaminergic transmission in this system may play a central role in many, if not all, addictions [54]. Animal studies show a dose-dependent increase in DA in response to alcohol in the NAc, indirectly mediated *via* the VTA. An increase in DA in the NAc occurs in conditioned animals in anticipation of ethanol administration. The local application of dopaminergic-specific neurotoxins in the NAc can lead to a reduction in ethanol consumption in alcohol-dependent rats. In human subjects, PET studies show a significant decrease in DA D_2_ receptor binding in alcoholics. Alcoholics also show reduced dopaminergic function that correlates with addiction severity. Despite these observations, DA agonists and antagonists have had limited success as treatments for alcoholism [55].

### Genetic variation in DRD2

The *DRD2* gene that encodes the D_2_ receptor has several well-characterized polymorphisms, some of which have been associated with diseases such as schizophrenia and dependence. The *Taq*IA SNP has been extensively studied in relation to alcohol misuse. There is, however, little agreement whether it is [56-64] or is not [65-69] associated with alcohol dependence. This work is further complicated by some studies being limited to special populations [66, 68], limited to a single gender [61, 64], or broadened to additional psychiatric disorders [58, 60, 64]. Meta-analyses also disagree, with some finding an association with alcohol dependence [70, 71] and others not [72]. The *Taq*IA story was further complicated when it was found that the SNP, which is ca. 9 kbp downstream of the last exon of *DRD2*, is in fact in the coding sequence of a poorly characterized neighboring gene, ankyrin-repeat containing kinase 1 (*ANKK1*) [73]. Work in this area continues.

## CURRENT TRENDS IN METHAMPHETAMINE USE AND ABUSE

Abuse of methamphetamine (METH) is a growing international public health problem with an estimated 35 million users worldwide, including countries like Canada, China, Japan, Mexico, and USA [74]. It is thought that over half of the world’s METH consumers reside in Southeast Asia. In Mexico, the number of people admitted to treatment for psychostimulant addiction from 3% in 1996 to 20% in 2006. METH is the most commonly synthesized illegal drug in the United States and has been cited by law enforcement officials as the leading cause of criminal problems in the country. A 2006 survey showed that 5.8% of Americans aged 12 years or older used METH at least once [75]. There have been substantial increases in METH-related emergency room admissions at hospitals in the Southwest of the USA.

After taking the drug, users experience a sense of euphoria, increased productivity, hypersexuality, decreased anxiety and increased energy. These effects can last for several hours. METH abuse is also associated with a number of negative which include acute toxicity, altered behavioral and cognitive functions, and neurological damage [76]. Ingestions of large doses of the drug can also cause more serious consequences that include life-threatening hyperthermia, renal and liver failure, cardiac arrhythmias, heart attacks, cerebrovascular hemorrhages, strokes and seizures. Chronic abuse of METH contributes to anxiety, depression, aggressiveness, social isolation, psychosis, mood disturbances, and psychomotor dysfunction. Withdrawal from METH can produce anhedonia, irritability, fatigue, impaired social functioning, and intense craving for the drug [76]. Neuroimaging studies have revealed METH-induced neurodegenerative changes in the brains of human addicts [77]. These include persistent decreases in the levels of dopamine transporters (DAT) in various brain. Structural magnetic resonance imaging (MRI) studies in METH addicts have documented substantial morphological changes in their brains [78].

Studies in animal models have reported that METH can cause depletion of dopamine, serotonin, and of their metabolites in the brain [79]. These abnormalities are associated with marked decreases in the DA and 5-HT transporters in various brain regions. These abnormalities are thought to be related to the production of oxygen-based radicals including superoxide radicals, hydrogen peroxide, and hydroxyl radicals. Damage to mitochondria and abnormal metabolism of other reactive compounds might also play a role in causing METH-induced damage in monoaminergic terminals [79]. Some of the damage are prevented by pretreatment with dopaminergic receptor blockers and trophic factors such as GDNF or BMP7 [80]. Another process that has shown to be protective involves administration of low doses for METH that are not toxic, suggesting that small doses of the drug can trigger molecular and cellular changes that render the brain refractory to its pro-oxidant properties [81]. If we can project this idea to the human condition, this process might explain why drug addicts do not develop signs and symptoms of Parkinsonism.

In summary, METH addiction is a major neuropsychiatric problem. Research is under way in order to understand the basic mechanisms involved in switching from exposure to drug to being addicted to METH. These studies involve behavioral, cellular and molecular neurobiological approaches. It is hoped that understanding of the pathways involved will lead to better treatment approaches to the clinical population of METH addicted individuals.

## TIME TO TAKE A CLOSER LOOK AT MDMA USE/ABUSE

MDMA became a popular drug in the USA in the 1970s and its use rapidly spread to Europe. Initially, drug use was associated with "raves" and the youth dance culture and in the early 1990s reports of excessive use were restricted to case studies [82-85]. Early surveys suggested that use was limited [86, 87] and the seeming ability to control intake led to the belief that the drug had limited abuse potential [88, 89] did “not seem addictive” [90] and had “almost no potential to lead to dependence” [91]. In 1995, the Monitoring the Future survey in the USA indicated an increase in ecstasy use among high school students. subsequent surveys indicated an increase in prevalence rates through to 2001 followed by a decrease. Current prevalence rates (2007) are at about 4% of high school students in the USA. The pattern of ecstasy use also appears to have changed quite considerably in more recent years. While most users consume MDMA relatively infrequently, increasingly the data indicate that many users consume MDMA frequently and in large amounts and some users met criteria for abuse and/or dependence as measured by DSM. The changes being observed in MDMA use and abuse are reminiscent of those that were seen with cocaine in the 1980s. Cocaine was also not initially considered to be “addictive” [92], a conclusion that we now know is not true. Some cocaine users consume the drug in a compulsive manner that characterizes abuse. MDMA users have also been classified as either novice or light users, moderate users or heavy users based upon either length of time of use, number of pills typically ingested per use or total lifetime use. When one examines the use patterns, it becomes apparent that heavy users take more pills on each occasion [93-96]. Specifically, the number of tablets usually taken and the largest number of tablets ever taken on one occasion was largest in the subjects classified as heavy MDMA users.

There is considerable controversy about the extent of short- and long-term consequences of MDM use and abuse. We have several decades of preclinical research that has investigated self-administration and other models of drug abuse. This knowledge and paradigm development is being applied to the study of MDMA abuse to ultimately understand the neurobiological mechanisms and the consequences of use and abuse of this drug.

## References

[1] Koob GF, Volkow ND (2010). Neurocircuitry of addiction. Neuropsychopharmacology.

[2] Uhl GR, Drgon T, Johnson C, Li C-Y, Contoreggi C, Hess J, Naiman D, Liu QR (2008). Molecular genetics of addiction and related heritable phenotypes: genome-wide association approaches identify “connectivity constellation” and drug target genes with pleiotropic effects. Ann. N.Y. Acad. Sci.

[3] Onaivi ES (2009). Cannabinoid receptors in brain: pharmacogenetics, neuropharmacology, neurotoxicology, and potential therapeutic applications. Int. Rev. Neurobiol.

[4] Onaivi ES, Leonard CM, Ishiguro H, Zhang P-W, Lin Z, Akinshola BE, Uhl GR (2002). Endocannabinoids and cannabinoid receptor genetics. Prog. Neurobiol.

[5] Karsak M, Cohen-Solal M, Freudenberg J, Ostertag A, Morieux C, Kornak U, Essig J, Erxlebe E, Bab I, Kubisch C, de Vernejoul MC, Zimmer A (2005). Cannabinoid receptor type 2 gene is associated with human osteoporosis. Hum. Mol. Genet.

[6] Sipe JC, Arbour N, Gerber A, Beutler E (2005). Reduced endocannabinoid immune modulation by a common cannabinoid 2 (CB2) receptor gene polymorphism: possible risk for autoimmune disorders. J. Leukoc. Biol.

[7] Lu AT, Ogdie MN, Järvelin MR, Moilanen IK, Loo S-K, McCracken JT, McGough JJ, Yang M-H, Peltonen L, Nelson SF, Cantor RM, Smalley SL (2008). Association of the cannabinoid receptor gene (CNR1) with ADHD and post-traumatic stress disorder. Am. J. Med. Genet. B Neuropsychiat. Genet.

[8] Cota D, Marsicano G, Lutz B, Vicennati V, Stalla GK, Pasquali R, Pagotto U (2003). Endogenous cannabinoid system as a modulator of food intake. Int. J. Obes.

[9] Jesudason D, Wittert G (2008). Endocannabinoid system in food intake and metabolic regulation. Curr. Opin. Lipidol.

[10] Serra G, Fratta W (2007). A possible role for the endocannabinoid system in the neurobiology of depression. Clin. Pract. Epidemiol. Ment. Health.

[11] Onaivi ES, Ishiguro H, Gong J-P, Patel S, Perchuk A, Meozzi PA, Myers L, Mora Z, Tagliaferro P, Gardner E, Brusco A, Akinshola BE (2006). Discovery of the presence and functional expression of cannabinoid CB2 receptors in brain. Ann. N.Y. Acad. Sci.

[12] Palomo T, Kostrzewa RM, Beninger RJ, Archer T (2007). Genetic variation and spared biological susceptibility underlying comorbidity in neuropsychiatry. Neurotox. Res.

[13] Mathers C, Vos T, Stevenson C (1999). The burden of injury and disease in Australia.

[14] WHO (2004). Global Status Report on Alcohol.

[15] AIHW (2002). 2001 National Drug Strategy Household Survey: First
results. Drug Statistics Series.

[16] Collins DJ, Lapsley HM (2002). Counting the cost: estimates of the social costs of drug abuse in Australia in 1998-9.

[17] AIHW (2003). Statistics on drug use in Australia 2002.

[18] Harper CG, Kril JJ, Hunt WA, Nixon SJ (1993). Neuropathological changes in alcoholics. Alcohol-Induced Brain Damage; NIH Publications: Rock.

[19] Kril JJ, Harper CG (1989). Neuronal counts from four cortical regions of alcoholic brains. Acta Neuropathol. (Berlin).

[20] Kril JJ, Halliday GM, Svoboda MD, Cartwright H (1997). The cerebral cortex is damaged in chronic alcoholics. Neuroscience.

[21] Saunders JB, Latt N, Hayes PC (1993). Epidemiology of alcoholic liver disease. Clinical Gastroenterology.

[22] Dodd PR, Williams SH, Gundlach AL, Harper PAW, Healy PJ, Dennis JA, Johnston GAR (1992). Glutamate and γ-aminobutyric acid neurotransmitter systems in the acute phase of maple syrup urine disease and citrullinemia encephalopathies in newborn calves. J. Neurochem.

[23] Crews FT, Buckley T, Dodd PR, Ende G, Foley N, Harper C, He J, Innes D, Loh E-W, Pfefferbaum A, Zou J, Sullivan EV (2005). Alcoholic neurobiology: changes in dependence and recovery. Alcohol Clin. Exp. Res.

[24] Koob GF, Le Moal M (2001). Drug addiction, dysregulation of reward, and allostasis. Neuropsychopharmacology.

[25] Koob GF (2003). Alcoholism: allostasis and beyond. Alcohol Clin. Exp. Res.

[26] Gulya K, Grant KA, Valverius P, Hoffman PL, Tabakoff B (1991). Brain regional specificity and time-course of changes in the NMDA receptor-ionophore complex during ethanol withdrawal. Brain Res.

[27] Snell LD, Tabakoff B, Hoffman PL (1993). Radioligand binding to the N-methyl-D-aspartate receptor/ionophore complex: alterations by ethanol *in vitro* and by chronic *in vivo* ethanol ingestion. Brain Res.

[28] Haugbol SR, Ebert B, Ulrichsen J (2005). Upregulation of glutamate receptor subtypes during alcohol withdrawal in rats. Alcohol Alcohol.

[29] Sircar R, Sircar D (2006). Repeated ethanol treatment in adolescent rats alters cortical NMDA receptor. Alcohol.

[30] Devaud LL, Morrow AL (1999). Gender-selective effects of ethanol dependence on NMDA receptor subunit expression in cerebral cortex, hippocampus and hypothalamus. Eur. J. Pharmacol.

[31] Darstein MB, Landwehrmeyer GB, Feuerstein TJ (2000). Changes in NMDA receptor subunit gene expression in the rat brain following withdrawal from forced long-term ethanol intake. Naunyn-Schmiedeberg s Arch. Pharmacol.

[32] Devaud LL, Alele P (2004). Differential effects of chronic ethanol administration and withdrawal on γ-aminobutyric acid type A and NMDA receptor subunit proteins in male and female rat brain. Alcohol Clin. Exp. Res.

[33] Nixon K, Hughes PD, Amsel A, Leslie SW (2004). NMDA receptor subunit expression after combined prenatal and postnatal exposure to ethanol. Alcohol Clin. Exp. Res.

[34] Nelson TE, Ur CL, Gruol DL (2005). Chronic intermittent ethanol exposure enhances NMDA-receptor-mediated synaptic responses and NMDA receptor expression in hippocampal CA1 region. Brain Res.

[35] Michaelis EK, Freed WJ, Galton N, Foye J, Michaelis ML, Phillips I, Kleinman JE (1990). Glutamate receptor changes in brain synaptic membranes from human alcoholics. Neurochem. Res.

[36] Freund G, Anderson KJ (1996). Glutamate receptors in the frontal cortex of alcoholics. Alcohol Clin. Exp. Res.

[37] Ridge JP, Ho AM.-C, Innes DJ, Dodd PR (2008). The expression of NMDA receptor subunit mRNA in human chronic alcoholics. Ann. NY Acad. Sci.

[38] Ridge JP, Dodd PR, Paley BF, Warfield TE (2008). Glutamate and ethanol: the role of ionotropic NMDA glutamate receptors in alcoholism. In: Amino Acid Receptor Research.

[39] Ridge JP, Dodd PR (2009). Cortical NMDA receptor expression in human chronic alcoholism: influence of the *TaqIA* allele of ANKK1. Neurochem. Res.

[40] Ridge JP, Ho AM-C, Dodd PR (2009). Sex differences in NMDA receptor expression in human alcoholics. Alcohol Alcohol.

[41] Arstad E, Platzer S, Berthele A, Pilowsky LS, Luthra SK, Wester HJ, Henriksen G (2006). Towards NR2B receptor selective imaging agents for PET — synthesis and evaluation of N-[^11^C]-(2-methoxy)benzyl (E)-styrene-, 2-naphthyl- and 4-trifluoro-methoxyphenylamidine. Bioorg. Med. Chem.

[42] Stone JM, Erlandsson K, Ärstad E, Bressan RA, Squassante L, Teneggi V, Ell PJ, Pilowsky LS (2006). Ketamine displaces the novel NMDA receptor SPET probe [^123^I]CNS- 1261 in humans *in vivo*. Nucl. Med. Biol.

[43] Matsumoto R, Haradahira T, Ito H, Fujimura Y, Seki C, Ikoma Y, Maeda J, Arakawa R, Takano A, Takahashi T, Higuchi M, Suzuki K, Fukui K, Suhara T (2007). Measurement of glycine binding site of *N*-methyl-D-asparate receptors in living human brain using 4-acetoxy derivative of L-703, 717, 4-Acetoxy-7-chloro-3-[3-(4-[^11^C] methoxybenzyl) phenyl]-2(^1^H)-quinolone (AcL703) with positron emission tomography. Synapse.

[44] Liu J, Lewohl JM, Dodd PR, Randall PK, Harris RA, Mayfield RD (2004). Gene expression profiling of individual cases reveals consistent transcriptional changes in alcoholic human brain. J. Neurochem.

[45] Liu J, Lewohl JM, Harris RA, Iyer VR, Dodd PR, Randall PK, Mayfield RD (2006). Patterns of gene expression in the frontal cortex discriminate alcoholic from nonalcoholic individuals. Neuropsychopharmacology.

[46] Etheridge N, Lewohl JM, Mayfield RD, Harris RA, Dodd PR (2009). Synaptic proteome changes in the superior frontal gyrus and occipital cortex of the alcoholic brain. Proteomics Clin. Appl.

[47] Foley PF, Loh EW, Innes DJ, Williams SM, Tannenberg AEG, Harper CG, Dodd PR (2004). Association studies of neurotransmitter gene polymorphisms in alcoholic Caucasians. Ann. NY Acad. Sci.

[48] Bergeson SE, Berman AE, Dodd PR, Edenberg HJ, Hitzemann RJ, Lewohl JM, Lodowski KH, Sommer W (2005). Expression profiling in alcoholism research. Alcohol. Clin. Exp. Res.

[49] Dodd PR, Buckley ST, Eckert AL, Foley PF, Innes DJ (2006). Genes and gene expression in the brains of human alcoholics. Ann. N.Y. Acad. Sci.

[50] Ponomarev I, Schafer GL, Blednov YA, Williams RW, Iyer VR, Harris RA (2004). Convergent analysis of cDNA and short oligomer microarrays, mouse null mutants and bioinformatics resources to study complex traits. Genes Brain Behav.

[51] Edenberg HJ (2007). The genetics of alcohol metabolism: role of alcohol dehydrogenase and aldehyde dehydrogenase variants. Alcohol Res. Health.

[52] Ghaemmaghami S, Huh WK, Bower K, Howson RW, Belle A, Dephoure N, O’Shea EK, Weissman JS (2003). Global analysis of protein expression in yeast. Nature.

[53] Di Chiara G (1997). Alcohol and dopamine. Alcohol Health Res. World.

[54] Pierce RC, Kumaresan V (2006). The mesolimbic dopamine system: the final common pathway for the reinforcing effect of drugs of abuse? Neurosci. Biobehav. Rev.

[55] Tupala E, Tiihonen J (2004). Dopamine and alcoholism: neurobiological basis of ethanol abuse. Prog. Neuropsychopharmacol. Biol. Psychiatry.

[56] Noble EP, Syndulko K, Fitch RJ, Ritchie T, Bohlman MC, Guth P, Sheridan PJ, Montgomery A, Heinzmann C, Sparkes RS, Kenneth B (1994). D2 dopamine receptor TaqI A alleles in medically ill alcoholic and nonalcoholic patients. Alcohol Alcohol.

[57] Ishiguro H, Arinami T, Saito T, Akazawa S, Enomoto M, Mitushio H, Fujishiro H, Tada K, Akimoto Y, Mifune H, Shioduka S, Hamaguchi H, Toru M, Shibuya H (1998). Association study between the -141C Ins/Del and TaqI A polymorphisms of the dopamine D2 receptor gene and alcoholism. Alcohol. Clin. Exp. Res.

[58] Lu RB, Lee JF, Ko HC, Lin WW (2001). Dopamine D2 receptor gene (DRD2) is associated with alcoholism with conduct disorder. Alcohol Clin. Exp. Res.

[59] Madrid GA, MacMurray J, Lee JW, Anderson BA, Comings DE (2001). Stress as a mediating factor in the association between the DRD2 TaqI polymorphism and alcoholism. Alcohol.

[60] Young RM, Lawford BR, Noble EP, Kann B, Wilkie A, Ritchie T, Arnold L, Shadforth S (2002). Harmful drinking in military veterans with post-traumatic stress disorder: association with the D2 dopamine receptor A1 allele. Alcohol Alcohol.

[61] Hallikainen T, Hietala J, Kauhanen J, Pohjalainen T, Syvalahti E, Salonen JT, Tiihonen J (2003). Ethanol consumption and DRD2 gene TaqI a polymorphism among socially drinking males. Am. J. Med. Genet. A.

[62] Berggren U, Fahlke C, Aronsson E, Karanti A, Eriksson M, Blennow K, Thelle D, Zetterberg H, Balldin J (2006). The taqI DRD2 A1 allele is associated with alcohol-dependence although its effect size is small. Alcohol Alcohol.

[63] Dick DM, Wang JC, Plunkett J, Aliev F, Hinrichs A, Bertelsen S, Budde JP, Goldstein EL, Kaplan D, Edenberg HJ, Nurnberger J, Hesselbrock V, Schuckit M, Kuperman S, Tischfield J, Porjesz B, Begleiter H, Bierut LJ, Goate A (2007). Family-based association analyses of alcohol dependence phenotypes across DRD2 and neighboring gene ANKK1. Alcohol Clin. Exp. Res.

[64] Ponce G, Hoenicka J, Jimenez-Arriero MA, Rodriguez-Jimenez R, Aragues M, Martin-Sune N, Huertas E, Palomo T (2008). DRD2 and *ANKK1* genotype in alcohol-dependent patients with psychopathic traits: association and interaction study. Br. J. Psychiatry.

[65] Gelernter J, O’Malley S, Risch N, Kranzler HR, Krystal J, Merikangas K, Kennedy JL, Kidd KK (1991). No association between an allele at the D2 dopamine receptor gene (DRD2) and alcoholism. JAMA.

[66] Chen CH, Chien SH, Hwu HG (1996). Lack of association between TaqI A1 allele of dopamine D2 receptor gene and alcohol-use disorders in atayal natives of Taiwan. Am. J. Med. Genet. C Sem. Med. Genet.

[67] Matsushita S, Muramatsu T, Murayama M, Nakane J, Higuchi S (2001). Alcoholism, ALDH2*2 allele and the A1 allele of the dopamine D2 receptor gene: an association study. Psychiatry Res.

[68] Shaikh KJ, Naveen D, Sherrin T, Murthy A, Thennarasu K, Anand A, Benegal V, Jain S (2001). Polymorphisms at the DRD2 locus in early onset alcohol dependence in the Indian population. Addict. Biol.

[69] Yang BZ, Kranzler HR, Zhao H, Gruen JR, Luo X, Gelernter J (2007). Association of haplotypic variants in DRD2, ANKK1, TTC12 and NCAM1 to alcohol dependence in independent case control and family samples. Hum. Mol. Genet.

[70] Noble EP (2003). D2 dopamine receptor gene in psychiatric and neurologic disorders and its phenotypes. Am. J. Med. Genet. B Neuropsychiat. Genet.

[71] Young RM, Lawford BR, Nutting A, Noble EP (2004). Advances in molecular genetics and the prevention and treatment of substance misuse: implications of association studies of the A1 allele of the D2 dopamine receptor gene. Addict. Behav.

[72] Munafò MR, Matheson IJ, Flint J (2007). Association of the DRD2 gene Taq1A polymorphism and alcoholism: a meta-analysis of case-control studies and evidence of publication bias. Mol. Psychiatry.

[73] Neville MJ, Johnstone EC, Walton RT (2004). Identification and characterization of *ANKK1*: a novel kinase gene closely linked to DRD2 on chromosome band 11q23.1. Hum. Mutat.

[74] UNODC (2007). World Drug Report Volume 1. Analysis.

[75] SAMHSA (2007). The NSDUH Report: Methamphetamine Use.

[76] Gold MS, Kobeissy FH, Wang KK, Merlo LJ, Bruijnzeel AW, Krasnova IN, Cadet JL (2009). Methamphetamine- and trauma-induced brain injuries: comparative cellular and molecular neurobiological substrates. Biol. Psychiatry.

[77] Volkow ND, Chang L, Wang G-J, Fowler JS, Franceschi D, Sedler M, Gatley SJ, Miller E, Hitzemann R, Ding YS, Logan J (2001). Loss of dopamine transporters in methamphetamine abusers recovers with protracted abstinence. J. Neurosci.

[78] Chang L, Ernst T, Speck O, Patel H, DeSilva M, Leonido-Yee M, Miller EN (2002). Perfusion MRI and computerized cognitive test abnormalities in abstinent methamphetamine users. Psychiatry Res.

[79] Krasnova IN, Cadet JL (2009). Methamphetamine toxicity and messengers of death. Brain Res. Rev.

[80] Chou J, Luo Y, Kuo C-C, Powers K, Shen H, Harvey BK, Hoffer BJ, Wang Y (2008). Bone morphogenetic protein-7 reduces toxicity induced by high doses of methamphetamine in rodents. Neuroscience.

[81] Cadet JL, McCoy MT, Cai N-S, Krasnova IN, Ladenheim B, Beauvais G, Wilson N, Wood W, Becker KG, Hodges AB (2009). Methamphetamine preconditioning alters midbrain transcriptional responses to methamphetamine-induced injury in the rat striatum. PLoS One.

[82] McGuire P, Fahy T (1991). Chronic paranoid psychosis after misuse of MDMA (“ecstasy”). Br. Med. J.

[83] Winstock AR (1991). Chronic paranoid psychosis after misuse of MDMA. Br. Med. J.

[84] Shearman JD, Chapman RW, Satsangi J, Ryley NG, Weatherhead S (1992). Misuse of ecstasy. Br. Med. J.

[85] Gorard DA, Davies SE, Clark ML (1992). Misuse of ecstasy. Br. Med. J.

[86] Peroutka SJ, Newman H, Harris H (1988). subjective effects of 3, 4-methylenedioxymethamphetamine in recreational users. Neuropsychopharmacology.

[87] Cohen RS (1995). subjective reports on the effects of the MDMA (‘ecstasy’) experience in humans. Prog. Neuropsychopharmacol. Biol. Psychiatry.

[88] Solowij N, Hall W, Lee N (1992). Recreational MDMA use in Sydney: a profile of 'Ecstacy' users and their experiences with the drug. Br. J. Addict.

[89] Beck J, Rosenbaum M (1994). Pursuit of Ecstasy: The MDMA Experience.

[90] Henry JA (1992). Ecstasy and the dance of death. Br. Med. J.

[91] Hammersley R, Ditton J, Smith I, Short E (1999). Patterns of ecstasy use by drug users. Br. J. Criminol.

[92] Washington AM, Gold MS, Stimmel B Recent rends in cocaine abuse: A view from the national hotline 800-COCAINE. In: Cocaine: Pharmacology Addiction and Therapy.

[93] Scholey AB, Parrott AC, Buchanan T, Heffernan TM, Ling J, Rodgers J (2004). Increased intensity of Ecstasy and polydrug usage in the more experienced recreational Ecstasy/MDMA users: a WWW study. Addict. Behav.

[94] Verkes RJ, Gijsman HJ, Pieters MS, Schoemaker RC, de Visser S, Kuijpers M, Pennings EJ, de Bruin D, van de Wijngaart G, van Gerven JM, Cohen AF (2001). Cognitive performance and serotonergic function in users of ecstasy. Psychopharmacology.

[95] Fox HC, Parrott AC, Turner JJ (2001). Ecstasy use: cognitive deficits related to dosage rather than self-reported problematic use of the drug. J. Psychopharmacol.

[96] Parrott AC (2005). Chronic tolerance to recreational MDMA (3, 4-methylenedioxymethamphetamine) or Ecstasy. J. Psychopharmacol.

